# Emerging Complexity in CD4^+^T Lineage Programming and Its Implications in Colorectal Cancer

**DOI:** 10.3389/fimmu.2021.694833

**Published:** 2021-08-20

**Authors:** Daniel DiToro, Rajatava Basu

**Affiliations:** ^1^Brigham and Women’s Hospital, Boston, MA, United States; ^2^Harvard Medical School, Boston, MA, United States; ^3^Ragon Institute of MGH MIT and Harvard, Cambridge, MA, United States; ^4^Division of Molecular and Cellular Pathology, Department of Pathology, University of Alabama at Birmingham (UAB), Birmingham, AL, United States

**Keywords:** CD4^+^T cell, effector T cell, regulatory T cell (Treg), T follicular helper cell (Tfh), T follicular regulatory cell (Tfr), lineage programming, plasticity, colorectal carcinoma

## Abstract

The intestinal immune system has the difficult task of protecting a large environmentally exposed single layer of epithelium from pathogens without allowing inappropriate inflammatory responses. Unmitigated inflammation drives multiple pathologies, including the development of colorectal cancer. CD4^+^T cells mediate both the suppression and promotion of intestinal inflammation. They comprise an array of phenotypically and functionally distinct subsets tailored to a specific inflammatory context. This diversity of form and function is relevant to a broad array of pathologic and physiologic processes. The heterogeneity underlying both effector and regulatory T helper cell responses to colorectal cancer, and its impact on disease progression, is reviewed herein. Importantly, T cell responses are dynamic; they exhibit both quantitative and qualitative changes as the inflammatory context shifts. Recent evidence outlines the role of CD4^+^T cells in colorectal cancer responses and suggests possible mechanisms driving qualitative alterations in anti-cancer immune responses. The heterogeneity of T cells in colorectal cancer, as well as the manner and mechanism by which they change, offer an abundance of opportunities for more specific, and likely effective, interventional strategies.

## Introduction

Despite being exposed to billions of microbes and their products, the basal tone of a healthy gut immune system is overtly tolerogenic. A strong tolerogenic capacity is beneficial to the host. Inappropriate activation of gut immunity underlies multiple inflammatory diseases. Chronic inflammation carries additional risk: it is a key factor in the development and progression of colorectal carcinoma (CRC) (1). This suppression cannot be absolute, however. Overcoming it is critical for mounting responses to pathogens, and for developing effective anti-cancer immune responses. The capacity to switch between tolerogenic and inflammatory states is one of the most critical aspects of gut immunity. This delicate balance is orchestrated by counteracting classes of CD4^+^T cells.

Naïve CD4^+^T cells are pluripotent precursors that differentiate into phenotypically and functionally distinct subsets uniquely tailored to operate in a specific inflammatory context. The differentiation of naïve, antigen-inexperienced CD4^+^T cells is a multi-step process and represents the integration of qualitative and quantitative variations in diverse signaling events guiding their development (2). Rational exploitation of CD4^+^T cell differentiation and function represents a potentially powerful avenue for therapeutic intervention. A nuanced understanding of the molecular determinants guiding these processes is a prerequisite for designing effective and safe therapies. Recent evidence has challenged long held notions regarding the conceptual and functional organization of T cell subsets, and our understanding of the roles these cells play in health and disease. These advances have illuminated an increasingly complex web of overlapping transcriptional networks. Emerging patterns hint at an underlying simplicity that may instruct potential therapeutic strategies.

## CD4^+^T Cell Heterogeneity – A Historical Perspective

Heterogeneity among CD4^+^T cells was first revealed by Mossman and Coffman in 1986, with the identification of Th1 and Th2 cells (3). This groundbreaking work lead to a period of intensive investigation and rapid discovery. The signaling and transcriptional events guiding these cell fates were identified, leading to the concept of ‘master regulator’ transcription factors (4–6). Additional effector subsets, including Th17 and Th22 cells, and the molecular determinants guiding their development, were discovered (7–11). The manner in which these distinct effector populations modulate cellular processes at the site of inflammation was carefully scrutinized.

The possibility that CD4^+^T cells also suppress inflammation was first proposed in 1970 by Gershon and Kondo (12, 13). The field became mired in controversy, however, and was effectively abandoned. The identification of distinct functional subsets by Mossman and Coffman led to a re-examination of this putative role. In 1995, Shimon Sakaguchi conclusively demonstrated the existence of regulatory T cells (Tregs) (14).

The role of T cells in driving antibody responses was also re-examined. T cells were known to be required for germinal center formation and class switched affinity matured antibody responses since the 1960’s, but the nature of this interaction and the specific cells participating in it remained unknown (15). Following establishment of the Th1/Th2 paradigm by Mossman and Coffman, it was proposed that, while Th1 cells regulate peripheral cellular events, Th2 cells functioned to provide help to B cells. This inference was based on their production of interleukin 4 (IL-4), which was shown to promote B cell proliferation in 1982 (15). However, deletion of Th2 genes, including *IL4*, failed to reduce germinal center and total IgG levels. Identification of Treg cells by Sakaguchi effectively overturned the nascent Th1/Th2 paradigm, and suggested germinal centers could depend on an as yet undiscovered subset. By the late 2000’s it was understood that help to B cells was provided by a distinct functional subset of CD4^+^T cells, termed T follicular helpers (Tfh) (16). Recently, a suppressive counterpart to Tfh, known as T follicular regulatory cells (Tfr), were identified (17).

**Figure 1:**
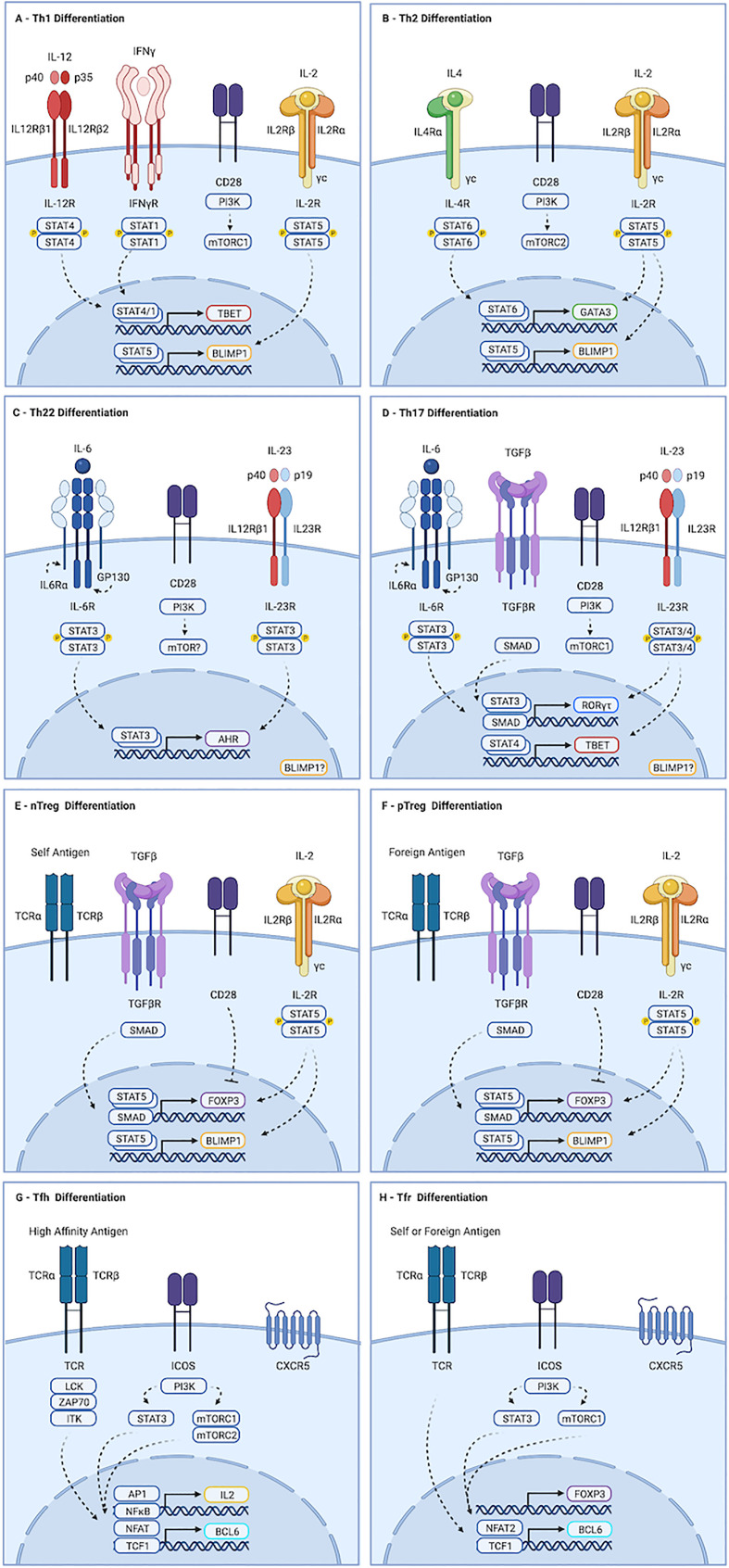
Molecular determinants guiding CD4^+^ T cell differentiation. **(A)** Th1 development is initiated by IL-12 mediated STAT4 dimerization, driving expression of *TBX21*. Activation of mTORC1, primarily by CD28, is also required. Maturation occurs in response to IL-12, and to STAT1 activation by autocrine IFN-γ. **(B)** Th2 differentiation is driven by IL-4, which promotes STAT6-dependent transcription of GATA3, and by mTORC2. **(C)** Th22 cells form in response to IL-6 driven STAT3 activation, leading to production of *AHR*. The contributions of mTORC1 and mTORC2 to this process remain unclear. **(D)** IL-6 in the presence of TGF-b-mediated SMAD activation and strong activation of mTORC1 drives transcription of *ROR-yt*, which primes cells to acquire a Th17 fate. Maturation occurs downstream of IL-23 mediated STAT3 activation. IL-23 and IL-1b can also promote STAT4-mediated induction of *TBX21* in Th17 cells, leading to production of IFN-γ and GM-CSF. **(E, F)** nTreg cells develop in the thymus following exposure to self-antigen. pTreg cells develop in the periphery in response to foreign antigen. Both require TGF-β and IL-2 to activate SMAD and STAT5 signaling, respectively, which drive transcription of *FOXP3*. While strong activation of AKT and mTOR favors effector cell development, weak induction favors regulatory cells. **(G)** Strong TCR stimulation and ICOS ligation by dendritic cells promotes Tfh differentiation. ICOS activates AKT, but also drives STAT3-mediated production of *TCF1*, which promotes expression of *BCL6*. Maturation requires continued TCR and ICOS stimulation by B cells. Recently activated cells fated to become Tfh produce IL-2. Signaling is largely paracrine, and drives STAT5 mediated induction of *BLIMP1*, a mutual antagonist of BCL6, in non-Tfh. **(H)** Events guiding Tfr differentiation overlap substantially with those of Tfh. Tfr are thought to be derived from FOXP3-positive precursors. As with Tfh, ICOS-mediated STAT3-dependent induction of *TCF1* promotes *BCL6* expression. However, Tfr appear to depend exclusively on mTORC1, whereas Tfh require both mTORC1 and mTORC2. Similarly, induction of CXCR5 in Tfr appears to require NFAT2, which is dispensable for Tfh development. Created with BioRender.com.

This heterogeneity of form and function is established *via* competing developmental signals driving lineage defining transcriptional events. The role of these cells, and the molecular determinants guiding their differentiation, are discussed below and summarized in **Figure 1**.

## Effector CD4^+^T Cell Subsets

### Th1

Th1 cells develop in response to intracellular pathogens (Type I responses). They promote the destruction of infected cells by inducing apoptosis and enhancing cytotoxic and phagocytic activity. Th1 cells also promote destruction of cancer cells, and drive much of the tissue damage seen during inflammation. Differentiation of Th1 cells is initiated by interleukin-12 (IL-12), a heterodimer consisting of a p35 and p40 subunit (**Figure 1A**) (18). Ligation with the IL-12 receptor, IL12R, drives STAT4-mediated expression of the transcription factor TBET (5, 19–21). Re-exposure to antigen and IL-12 at the site of inflammation induces maturation, allowing production of cytokines including interferon-*γ* (IFN-*γ*). Autocrine IFN-*γ* signaling further contributes to maturation of Th1 cells *via* STAT1-mediated stabilization of *TBET* (22).

### Th2

Type II responses to extracellular multicellular pathogens like helminths drive production of interleukin-4 (IL-4), which promotes STAT6-mediated transcription of *GATA3* and acquisition of a Th2 fate identity (**Figure 1B**) (6, 23). Peripheral maturation of Th2 cells permits secretion of a variety of cytokines, including IL-4 and interleukins 5 and 13 (IL-5, IL-13), which promote degranulation of eosinophils and mast cells. Dysregulated Th2 development this leads to hypersensitivity diseases, including asthma and allergy (24).

### Th17

Th17 cells promote responses to extracellular single cell pathogens (Type III responses). They recruit neutrophils and macrophages to the site of inflammation and stimulate phagocytosis of the invading microbes (25). Differentiation of Th17 cells is guided by the transcription factor ROR*γ*t, which is expressed in response to the cytokines TGF-β and interleukin 6 (IL-6) (**Figure 1D**) (7–11). Priming of Th17 cells by IL-6 up-regulates the IL-23 receptor (IL23R). Peripheral maturation of Th17 cells is driven by interleukin-23 (IL-23), a heterodimer composed of the IL-12p40 subunit complexed with a p19 subunit (26). IL-23 and IL1-β can activate STAT4 in Th17 cells, leading to induction of TBET and IFN-*γ*. Co-production of IFN-*γ* is pathogenic in many autoimmune and immune mediated diseases, though it is protective in anti-tumor responses (discussed in greater detail below).

### Th22

Th22 cells are critical regulators of epithelial barrier integrity and remodeling (27–30). Th22 cells secrete the cytokines interleukin-22 (IL-22) and tumor necrosis factor alpha (TNF-α), but do not produce IL-17A or IFN-*γ*. Development of Th17 cells requires STAT3 activation by IL-6 in the absence of TGF-β (**Figure 1C**). IL-23 enhances production of IL-22 from Th22 cells. Though no single lineage specifying transcription factor has been identified, aryl hydrocarbon receptor (AHR) is required for their optimal development. Th22 cells also express TBET and RORγt, albeit at levels below those seen in Th1 and Th17 cells, and deletion of these transcription factors reduces Th22 numbers.

### Tfh

Tfh cells orchestrate germinal center B cell responses. They are required for most class-switched affinity matured antibody responses (16). Strong antigenic stimulation and ICOS ligation by dendritic cells (DCs) drives expression of the transcription factor BCL6, the surface receptor PD-1, and the chemokine receptor CXCR5 (**Figure 1G**) (31–34). Primed cells, sometimes referred to as pre-Tfh, migrate to B cell follicles along a CXCL13 gradient. Maturation of Tfh cells occurs in response to sustained TCR and ICOS stimulation by B cells (31, 32, 35). Tfh develop in response to all major classes of pathogens. They are also seen in autoimmune diseases, and play physiologically relevant roles in response to some cancers (16). Abortive development of Tfh is seen even in response to organisms like Listeria monocytogenes that do not require or support germinal center reactions, suggesting early commitment to Tfh fate may be a universal feature of T cell activation (36).

## Regulatory CD4^+^T Cell Subsets

### nTregs & pTregs

CD4^+^T cells are also essential for maintaining tolerance to self-antigens, commensal microbes and dietary antigens (37). Tolerance to self-antigens is mediated by natural regulatory T cells (nTreg), which develop in the thymus in response to moderately-high affinity antigen (38–40). Treg cells specific to foreign antigens develop in the periphery (pTregs) (41–43). While strong induction of the PI3K-AKT-mTOR pathway by co-stimulation and cytokine-mediated activation of STAT3, STAT4, or STAT6 promote pro-inflammatory outcomes, Treg fate determination is favored by TGF-β-mediated SMAD activity, STAT5 activation downstream of interleukin-2 (IL-2), and weak PI3K-AKT-mTOR stimulation (**Figures 1E, F**) (44, 45). Development of Tregs requires the transcription factor FOXP3. Suppression of inflammation by Treg cells is mediated by contact-dependent mechanisms, including CTLA and PD-1 ligation, and secretion of the cytokine interleukin-10 (IL-10). Importantly, their influence often manifests in unpredictable ways: In many contexts, Treg cells are required for optimal inflammatory responses (46).

### Tfr

T follicular regulatory (Tfr) cells constrain germinal center (GC) processes (17, 47, 48). They develop in a wide range of inflammatory contexts, including infection, autoimmunity, and cancer. Tfr cells prevent production of auto-reactive antibodies and taper GC reactions during resolution of inflammation. As with Treg cells, the constraint provided by Tfr can also be required for optimal inflammatory responses (49, 50). Tfr are predominantly derived from nTreg cells, but can also develop from naïve precursors (47, 51, 52). The preponderance of naïve *versus* nTreg derived cells varies by tissue, with gut associated lymphoid tissues containing higher numbers of Tfr specific to foreign antigens and derived from naïve cells (53). Both BCL6 and FOXP3 are required for Tfr development, in parallel with their pro-inflammatory Tfh and suppressive Treg counterparts (**Figure 1H**) (47, 54). The molecular determinants guiding Tfr fate acquisition overlap substantially with that of Tfr, and include ICOS-mediated STAT3-dependent induction of TCF1, which promotes transcription of *BCL6* (55, 56). However, whereas NFAT2 is dispensable in Tfh, it is required by Tfr. Furthermore, while mTORC1 and mTORC2 contribute to Tfh development, Tfr appear to depend exclusively on mTORC1 (57, 58).

## Overlapping Transcriptional Networks

The historic progression of discoveries in the field of lymphocyte biology led to a model whereby one master regulator transcription factor is necessary and sufficient for one cell type. Master regulator transcription factors are commonly understood to be both necessary and sufficient for the acquisition of a cell fate. While this framework proved useful in identifying important transcriptional networks, further investigation revealed these factors are not sufficient for complete lineage programming and, in some cases, not absolutely required. For example, RORγt is insufficient for complete Th17 programming, Bcl6 is not sufficient for Tfh programming and ectopic Foxp3 expression confers only partial Treg identity (59, 60). Cooperation with additional transcription factors is necessary (61, 62).

Nor are these factors unique to specific populations. Indeed, there is substantial overlap in genetic programming between lymphocyte subsets. The Tfh compartment provides a useful illustration of this phenomenon. Tfh exhibit similar heterogeneity to that seen in non-Tfh effectors (63). During type I responses, Tfh cells express low levels of TBET and IFN-*γ* (31, 64). They express GATA3 and IL-4 during type 2 responses, and can produce IL-13 and IL-15 (65, 66). Tfh have also been shown to express RORγt and IL-17A (67–69). Production of these cytokines by Tfh guides isotype switching in B cells (70).

These transcriptional networks also regulate the function of regulatory cells. Tfr cells transiently express TBET during Type I responses. TBET, GATA3, and RORγt are expressed in a subset of FOXP3+ Treg cells termed effector regulatory T (eTreg) cells (48, 71). eTreg cells are enriched in peripheral tissues and are the primary mediators of suppressive functions. Expression is dependent on the local inflammatory context, correlates with the effector response, and is required to elicit optimal suppressive capacity. Conversely, some eTreg cells demonstrate compromised suppressor function and promote anti-tumor immunity, including in colorectal carcinoma (CRC) (72). This phenomenon, discussed in greater detail below, also appears dependent on expression of canonical effector transcription factors.

Their influence extends beyond CD4^+^T cells. TBET is often expressed in B cells, and is required for optimal antibody production during Type I responses (73, 74). Both innate lymphoid cells and invariant natural killer T cells express TBET, GATA3, or RORγt depending on the inflammatory environment (75, 76). Thus, rather than functioning as bona fide master regulators, it appears these proteins may overlay context-specific programming onto multiple lymphocyte lineages.

As traditional lines blur, others come into focus. BCL6 and BLIMP1, encoded by the gene *PRDM1*, are mutually antagonistic transcription factors. Tfh express BCL6, and effector cells produce BLIMP1 (77, 78). This bifurcation begins soon after activation. A limited and discrete subset of activated cells produce the cytokine IL-2 (79). These cells are marked by early expression of BCL6 and supply the Tfh compartment (**Figure 1G**) (80). IL-2 signaling at early time points is largely paracrine, inducing BLIMP1 in IL-2-negative cells *via* STAT5. BLIMP1 inhibits BCL6 and IL-2, reinforcing a non-Tfh fate, and collaborates with TBET and GATA3 to promote Th1 and Th2 development and function (**Figures 1A, B**) (81–84).

The role of IL-2, STAT5 and BLIMP1 in Th17 and Th22 cells is less clear. In mice, activation of STAT5 downstream of IL-2 inhibits Th17 development (45). In humans, however, IL-2 is crucial for optimal Th17 responses (85). *In vitro* primed murine Th17 cells express little to no BLIMP1 (86). Early studies crossing CD4-Cre or proximal Lck-Cre mice to *PRDM1* floxed mice, leading to deletion of *PRDM1* in the thymus, revealed colonic inflammation mediated by increased Th17 numbers, suggesting BLIMP1 opposes Th17 function (87). However, thymic deletion generates multiple developmental defects. Peripheral deletion of BLIMP1 using distal Lck-Cre mice leads to a reduction in Th17 numbers and amelioration of Th17-mediated inflammation (88). In this study, IL-23 was shown to mediate induction of BLIMP1 *via* STAT3, suggesting BLIMP1 may play a role in Th17 maturation (**Figure 1D**). Unfortunately, the role of BLIMP1 in Th22 cells remains largely unexamined. Th22 cells notwithstanding, this evidence suggests BCL6 and BLIMP1 mark pro-inflammatory cells that primarily support humoral *versus* cellular responses across multiple inflammatory contexts.

**Figure 2:**
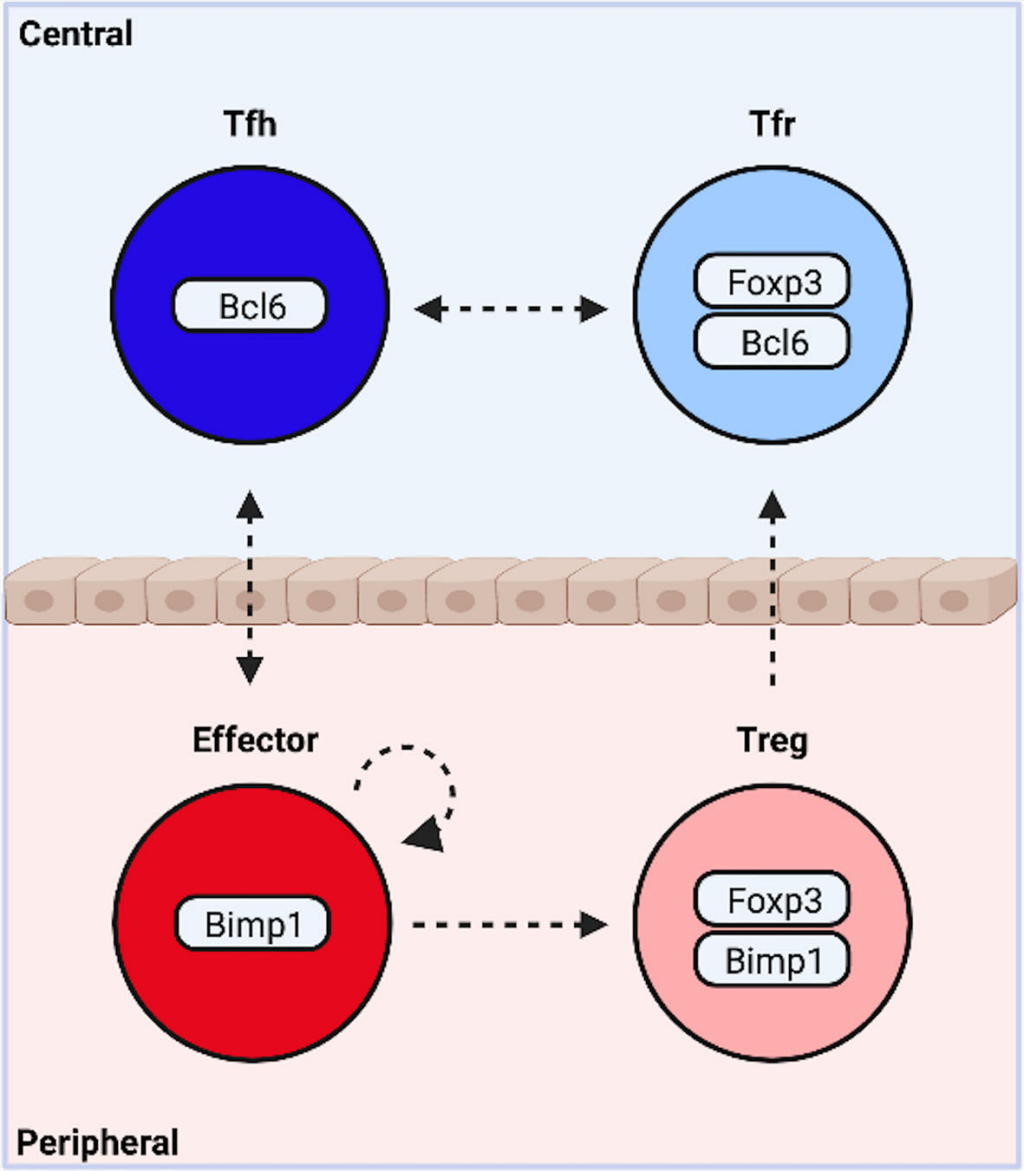
Functional Bifurcations Among CD4^+^ T Cells. Following activation, naïve cells are programmed to modulate central or peripheral processes. Similarly, activated cells either drive or suppress inflammation. These functional bifurcations are coincident and sufficiently independent to allow for the simultaneous generation of all four potential outcomes. Pro-inflammatory T follicular helper (Tfh) cells organize germinal center responses, while traditional non-Tfh effector subsets promote cellular responses at the site of inflammation. Both exist in mutual opposition with a suppressive counterpart. T follicular regulatory cells (Tfr) modify central events, while peripherally induced regulatory T cells (pTregs) suppress peripheral inflammation. The dynamic heterogeneity of CD4^+^ T cell responses may be due in part to plasticity between subsets (indicated by arrows). Created with BioRender.com.

Both Tfh and non-Tfh effector cells exist in mutual opposition with a FOXP3+ suppressive counterpart. Intriguingly, BLIMP1 is required for optimal production of IL-10 and suppression of peripheral inflammation by eTreg cells (71, 89, 90). Expression occurs downstream of TCR-mediated activation of IRF4, and STAT5 phosphorylation by IL-2 (**Figures 1E, F**) (87). In contrast, BCL6 is indispensable for Tfr. Thus BLIMP1 appears essential to most, and possibly all, peripheral subsets, while BCL6 is required by central, follicular T cells. It is therefore tempting to suggest the complexity of CD4^+^T cell differentiation may be collapsed into outcomes along two functional dimensions. One dimension describes a cooperative relationship between cells in distinct locations, the other an antagonistic relationship between cells occupying the same niche **(** **Figure 2** **)**.

There is reason to suspect this model may hold some validity. The conceptual organization is reflected in the underlying transcriptional programming, and is highly generalizable to different inflammatory settings. Indeed, these four subsets may be a necessary result of the both function and architecture of the adaptive immune system. The ubiquity of host-pathogen interactions and commensal microbial communities coupled with the destructive nature of immune responses necessitate a system capable of both driving and suppressing inflammation. The low copy number and exceptional diversity of receptor clonotypes necessitate localization in specialized tissues that permit deep sampling of the repertoire. The need to modulate events at the site of inflammation requires cell types that egress from these tissues, while complicated highly compartmentalized processes like germinal center reactions require cells dedicated to central events. Given this, Tfh, Tfr, Treg and effector cells may represent fundamental functional states, while overlapping transcriptional networks modify these core states to suit specific inflammatory settings, thereby increasing the diversity of potential outcomes.

## Plasticity of Effector & Regulatory CD4^+^T Cell Subsets

The transcriptional programs that guide these fate outcomes are not mutually exclusive, nor are they necessarily static. Lymphocyte phenotypes change at the population level as inflammatory responses mature. This is seen in multiple contexts, including the late emergence of distinct cytokine producing effector subsets, or the development of memory cells. These changes can be accomplished *via* two non-exclusive mechanisms; selective amplification of underlying heterogeneity, and the conversion of cells from one phenotype to another.

Data suggests the dynamic heterogeneity of effector responses may in part be due to lineage plasticity **(** **Figure 2** **)**. Naïve cells primed *in vitro* under conditions promoting Th1, Th2 or Th17 differentiation can acquire different phenotypes upon re-stimulation (91). Th17 cells appear to be particularly adept at acquiring the functions and phenotypes of other lineages (92–94). De novo co-expression of IFN-*γ* by Th17 cells occurs *in vivo* and represents a key source of IFN-*γ* in multiple pathologies. *In-vitro* generated Th17 cells can convert into IL-17A–negative IFN-*γ* producers in response to STAT4 activation downstream of IL-23 following adoptive transfer (95–97). At least one study utilizing IL-17A fate reporter mice suggests trans-differentiation into Th1 cells may also occur *in vivo* (97). TGF-β, a potent repressor of Th22 cells, can also induce AHR and IL-22 in Th17 cells (98). Co-expression of GATA3 and Th2 cytokines in Th17 cells is also documented (99).

Expression of TBET, GATA3, and RORγt, and their associated cytokines, by Treg and Tfh cells is variably described as plasticity in the literature. This terminology is somewhat controversial. Co-expression of canonical effector transcriptional modules is required for optimal function and may simply represent normal developmental maturation. De novo transition from one effector module to another *in vivo* has not been shown. However, it seems reasonable to consider pro-inflammatory eTreg cells in CRC an example of plasticity. While these cells do not fully extinguish FOXP3, they alter their core transcriptional networks and adopt a fundamentally different functional state. Certainly this represents meaningful functional plasticity, if not bona fide lineage conversion. Nevertheless, the role of plasticity in driving the heterogeneity seen within Treg and Tfh populations remains murky. Studies addressing the duration and stability of these states *in vivo* are needed.

More substantial evidence indicates plasticity between effector, Treg, Tfh and Tfr lineages may also occur **(** **Figure 2** **)**. nTreg cells supply the majority of the Tfr compartment. Some studies suggest Tfr may convert into Tfh *in vivo*, and Tfh can be converted into Tfr *in vitro* (53, 100–102). Fate mapping indicates former IL-17A-producing cells can transition into pTreg cells downstream of TGF-β-mediated induction of AHR (103). Lineage reporter mice also suggest Treg cells can lose FOXP3 and develop into pro-inflammatory ex-Tregs displaying Th1 or Th17 effector phenotypes (104, 105). Conversion of effector cells to Tfh appears negligible in many contexts. However, former IL-17A producing cells can exhibit a Tfh-like phenotype and guide IgA production in Peyer’s Patches (106). Similarly, while deletion of IL-2 producing Tfh precursors does not affect Th1 and Th2 numbers, it can lead to a reduction in Th17 cells (80). These findings suggest Tfh and Th17 development may be uniquely related. Peripheral Tfh-like cells may also indicate overlap between Tfh and effector lineages (107, 108). These cells exhibit qualities consistent with both effector and Tfh lineages, organize ectopic lymphoid tissues, and are capable of providing help to B cells. However, it remains unclear if they represent Tfh that migrated to the periphery, effectors that acquired a Tfh-like phenotype, or the *de novo* generation of an intermediate phenotype. Together these data suggest limited plasticity between Tfh, Tfr, Treg and effector cells is possible. Notably, interconversion between Tfh and Treg cells, and effector and Tfr cells, has not been observed, suggesting plasticity may be restricted along individual functional dimensions.

The cellular sources and molecular mechanisms underlying this apparent lineage plasticity remain uncertain. Many studies indicate mature Treg, Tfh and effector cell phenotypes are remarkably stable (109–113). In contrast, substantial evidence supports the existence of a window early in T cell differentiation in which activated cells maintain a state of pluripotency. Limiting dilution adoptive transfer experiments indicate single naïve CD4^+^T cells can give rise to both Tfh and effector cells (33). Recently activated cells exhibit epigenetic instability that is extinguished upon initiation of cell cycle progression and developmental maturation (114, 115). Furthermore, some cells transiently co-express multiple lineage programming transcription factors shortly after activation (116, 117). Indeed, this phenomenon complicates interpretation of lineage reporter experiments and may underlie results initially interpreted as supporting conversion of Treg cells to effectors (109, 110). Co-expression is likely mediated by convergent signaling events. Th17 development, in particular, exhibits substantial overlap with other lineages. TGF-β is required for Th17 and regulatory T cell development. STAT3 is required by Th17, Th22, Tfh and Tfr cells. STAT4 promotes IFN-*γ* production in both Th17 and Th1 cells. Thus plasticity between functional states may plausibly result from incomplete development following cell priming, and partial overlap between competing developmental pathways.

Caution, however, is warranted in interpreting data regarding cellular plasticity. Many studies utilize *in vitro* generated cells and adoptive transfer techniques. But *in vitro* polarized cells are not equivalent to mature *in vivo* effectors, and adoptive transfer into inflamed hosts may not reflect normal physiologic processes. Even *in vivo* experiments utilizing lineage reporter mice suffer from limitations. The fidelity with which a reporter gene indicates a given cell fate can be compromised, For example, while the vast majority of IL17A producers are Th17 cells, some Tfh produce IL17A, confounding efforts to address the relationship between these cells. In addition, transient expression can permanently activate a reporter construct without stable adoption of a cell fate. However, even with these limitations in mind, the abundance and diversity of data supporting plasticity strongly suggest it is both real and relevant to many physiologic and pathophysiologic contexts, including CRC.

## Colorectal Cancer

**Figure 3:**
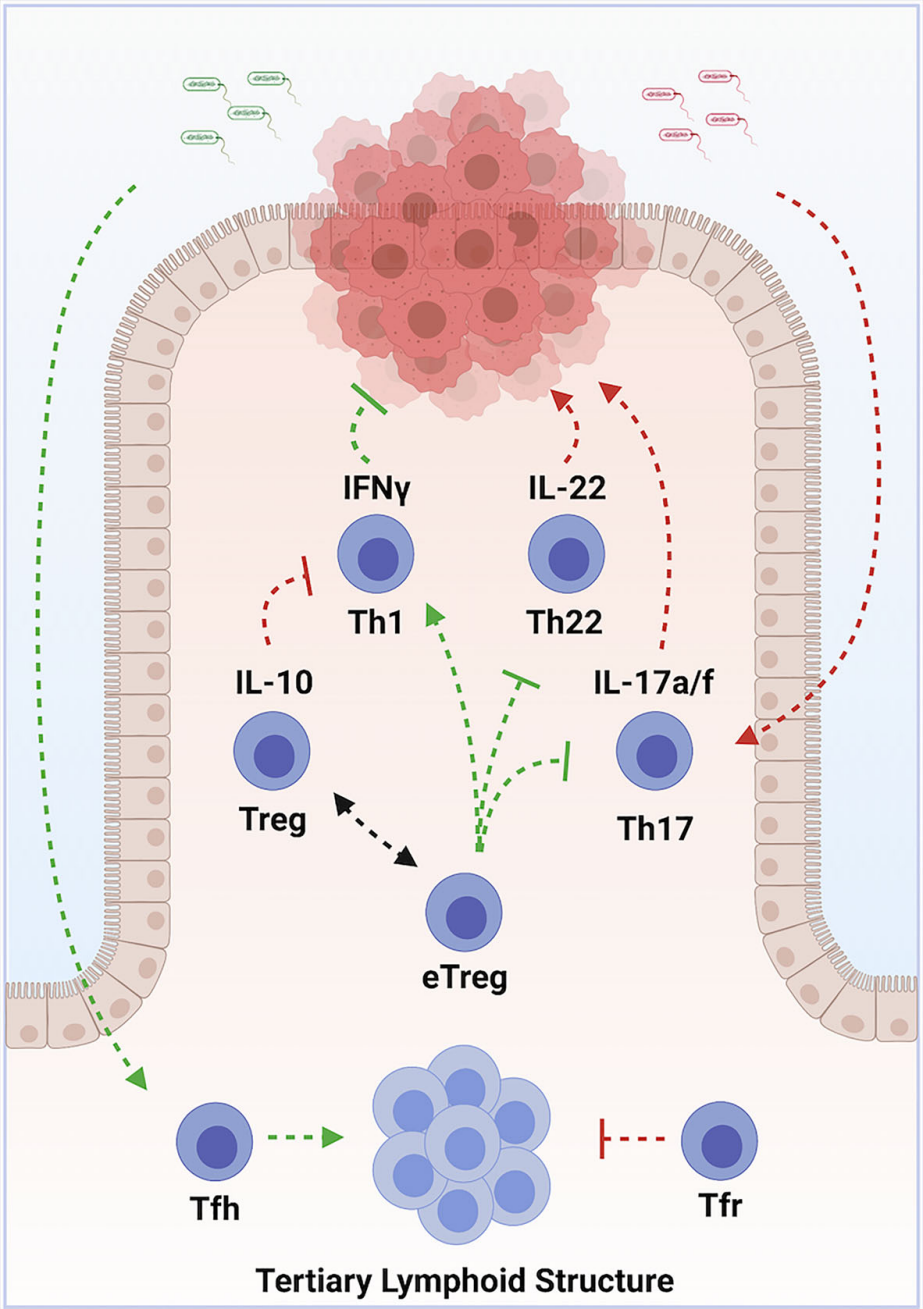
Multilayered roles of various subsets of CD4^+^ T Cells in Colorectal Carcinoma. Chronic inflammation, driven by Th17 cells in response to commensal organisms, promotes tumor development. Sustained exposure to IL-22, produced by Th22 cells, contributes to tumorigenesis. Th1 cells promote tumor cell destruction via production of IFN-γ. Treg cells oppose tumor development by suppressing chronic inflammation, but contribute to progression by opposing optimal tumor responses. Some types of pro-inflammatory eTreg cells, in contrast, promote tumor immune responses. Tumor colonization by protective commensal species drives accumulation of Tfh, which organize tertiary lymphoid structures. These structures enhance tumor immune responses and predict responses to chemo- and immune-therapeutics. Arrows indicate positive modulation; perpendicular lines indicate inhibitory relationships. Green indicates an overall anti-tumor effect, while red indicates an overall pro-tumorigenic effect. Created with BioRender.com.

Colorectal carcinoma (CRC) is the third most frequently diagnosed cancer in both men and women in the United States, with >140,000 cases diagnosed each year (CDC). It is also the third leading cause of cancer deaths, depriving >50,000 patients of their lives each year. CRC represents 98% of colonic cancers, and the WHO recognizes 6 distinct tumor subtypes. Most tumors develop as a result of sequential mutations driving progression along multiple potential pathways (118). Chronic inflammation is a well-recognized driver of tumorigenesis (1). Microbial dysbiosis is common in colorectal carcinoma, and may also contribute to tumorigenesis (119, 120). In the colon, Th1, Th17, Th22, pTreg and nTreg cell subsets exist in a state of dynamic equilibrium at epithelial barrier sites. Tfh additionally modulate colonic inflammation *via* the organization of ectopic lymphoid structures. Dysregulation of these cell populations can lead to chronic inflammation and dysbiosis. Immunotherapy therefore holds tremendous promise in treating CRC **(** **Figure 3** **)** (121).

## Role of Effector CD4^+^T Cell Subsets in Colorectal Cancer

Increased tumor infiltration by Th1 cells correlates with better prognosis (122, 123). This protection is likely mediated by the anti-proliferative, pro-apoptotic and anti-angiogenic actions of IFN-*γ*, as well as through enhanced recruitment of cytotoxic CD8 T cells (124). Th17 and Th22 cells, in contrast, are elevated in advanced disease and correlate with poor prognosis (125). Limited production of IL-22 can protect against genotoxic stress, but prolonged exposure drives uncontrolled proliferation of colonic epithelium, and promotes cancer stemness and chemo-resistance (126–129). IL-17A directly stimulates tumor growth and progression (130–132). IL-17A also stimulates angiogenesis *via* production of VEGF (133). Tumorigenic Th17 cells accumulate in response to IL-23, which is produced following microbial colonization of tumors due to barrier defects (134). Evidence indicates effector lineage plasticity may contribute to the pathogenesis of CRC. Th1-like IFN-*γ*+ Th17 cells exhibit potent anti-tumor properties (135, 136). In contrast, induction of IL-22 in Th17 cells downstream of TGF-β and AHR ligand promotes tumorigenesis (98).

While some microbial species promote tumorigenic Th17 cells, others predict enhanced responses to chemo- and immune-therapy (137–140). Colonization by protective organisms is associated with increased numbers of Tfh and the development of ectopic lymphoid structures (141). Accumulation of Tfh is associated with prolonged survival in humans (142). In mouse models, both Tfh and B cells are required for the protective effects conferred by these microbial species. Intriguingly, Tfr cells also accumulate at tumor sites, and may regulate Tfh functions (143).

## Role of Regulatory CD4^+^T Cell Subsets in Colorectal Cancer

Treg cells exhibit conflicting roles CRC. Preclinical and clinical studies indicate Treg cells suppress effector T cell-mediated immune responses to cancer (144, 145). Treg infiltration in CRC has been associated with tumor progression, lymphatic invasion and metastasis (146–148). However, eTregs, which are abundant in the intestine, can also promote anti-tumor immunity to, and induce regression of, intestinal cancers (149, 150). Indeed, tumor infiltrating Treg cells are associated with improved prognosis in many studies (72, 151–153).

These discordant results may be due to heterogeneity within the Treg compartment. During inflammatory responses, Treg cells can be divided into 3 main compartments; Suppressive CD45RA+ FOXP3-high naïve-like cells, suppressive CD45RA– FOXP3-high eTreg cells, and pro-inflammatory CD45RA– FOXP3-low eTreg cells. ROR-*γ*τ+ IL-17A+ FOXP3-high eTreg cells exhibit potent T cell suppression, but fail to restrain innate inflammation. They increase with tumor stage in human CRC, and promote tumor development in colitis-associated mouse models (154, 155). In contrast, FOXP3-low eTreg cells exhibit reduced T cell suppressive capacity and promote anti-tumor immunity (156, 157). Indeed, tumors harboring FOXP3-low eTreg cells that secrete IL-17A and/or IFN-*γ* are associated with significantly better prognosis (72). Tumors containing these cells exhibit increased expression of IL-12, has been speculated promote acquisition of this pro-inflammatory state. Cell lineage and target antigen may also influence this functional divide: While TCR sequences of Th17-like eTreg cells overlap with pTreg cells, Th1-like eTregs appear to be thymically derived (158).

## Targeting Subsets of CD4^+^T Cells in CRC: Therapeutic Implication

Treatment of CRC is guided by tumor stage and grade, but commonly involves surgical resection (159). Peri-operative chemotherapy is the standard of care for Stage III and IV tumors, and may be considered for stage II tumors. Established nearly two decades ago, Oxaliplatin, 5-fluorouracil and leucovorin (FOLFOX) still remains the first line regimen, although inhibition of VEGF or Ras signaling may offer statistically significant but limited improvement of outcomes in some cases. However, overall survival of localized, regional and metastatic CRC is only 91%, 72% and 13%, respectively (159). Therefore, additional therapeutic options are needed for therapeutic intervention.

Given the importance of T cells in modulating its pathophysiology, therapeutic approaches targeting lymphocyte function represent a promising addition to CRC treatment regimens. Defective mismatch repair (dMMR) leads to an abundance of tumor neoantigens. dMMR tumors are heavily infiltrated by Th1 cells and confer improved prognosis (122). Furthermore, dMMR tumors commonly exhibit elevated expression of PD-1 and PD-L1. Increased neoantigen burden and PD-1/PD-L1 mediated immune evasion suggest these tumors may be susceptible to checkpoint inhibition. Indeed, early trials examining the efficacy of PD-1 inhibition in dMMR tumors generated promising results (160). However, dMMR tumors are more commonly identified in earlier stages, and represent only 3-6% of advanced cases. Interventions targeting lymphocyte functions independent of checkpoint blockade are likely required for therapeutic efficacy in the majority of tumors.

Three general approaches to targeting CD4^+^T cells could be considered for CRC therapy: **A.** Direct inhibition CD4^+^T cell-derived tumor promoting factors. **B.** Interventions manipulating heterogeneity within CD4^+^T cell functional categories (Th1, Th17, Th22, Treg, eTreg, Tfh, Tfr etc.). **C.** Manipulation of the colonic microbiota. Importantly, successful implementation of each approach is currently impeded by an incomplete understanding of the relevant biology. Limited insight confers a limited capacity to intervene.

### Direct Inhibition of CD4^+^T Cell-Derived Tumor Promoting Factors

Direct inhibition of effector cytokines known to drive tumor progression may improve outcomes. The suppressive cytokine IL-10 is a potential target to elicit a robust anti-tumor immunity. Serum IL-10 is positively correlated with tumor stage and negatively correlated with prognosis in CRC patients (161, 162). IL-10 is increased in the CRC microenvironment, and IL-10RA levels correlate with KI67 staining (163). IL-10 blocking antibodies drive accumulation of tumor-infiltrating lymphocytes (TILs), release of granzyme B, and tumor cell necrosis in an *in vitro* human CRC culture system (164). Systemic blockade of IL-10 or IL-10RA, however, carries substantial risk. Targeted approaches may be required. Intra-tumor injection of lentivral vectors encoding IL-10 shRNA reduces IL-10 expression and potentiates bone marrow derived dendritic cell vaccine efficacy in a mouse model of CRC (165). IL-10 shRNA alone was not effective, and IL-10 production by T cells was unaffected. Caution, however, is warranted. Mouse models indicate IL-10 can actually augment cancer responses. Indeed, exogenous IL-10 is being investigated as a therapeutic option in multiple cancer types, including CRC (166, 167). Identification of the specific cellular sources of IL-10 that inhibit tumor immunity and targeted suppression of IL-10 production in those cells, or inhibition of IL-10RA signaling in tumor cells, may offer improved safety and efficacy. Regardless, the seemingly contradictory findings surrounding IL10 make it abundantly clear that our understanding of the underlying biology is profoundly limited. It is difficult to predict outcome of actions without an accurate model of what is being acted upon.

Given the roles of Th17 and Th22 cells in promoting tumor development, IL-17A, IL-17F, and IL-22 are also promising targets in CRC. Deletion of Il17a or Il17f reduces tumor development in an APC-driven mouse model of CRC (130, 168). Blockade of the IL-17/IL-17RA axis may also improve the efficacy of anti-VEGF therapies. Anti-IL22 antibodies inhibit CRC cell proliferation *in vitro* (169). Gene therapy designed to drive expression of IL-22BP, a secreted binding protein that inhibits IL-22 signaling, reduces tumor burden in mice (170). Again, caution is warranted as some studies indicate disruption of Th17 and Th22 cell function can promote tumor development and progression (171). The cause of these disparate outcomes is not fully understood, but may relate to the specific mechanism of CRC pathogenesis and the role of T cells in promoting appropriate *versus* chronic, dysregulated inflammatory responses. Further elucidation of the role of these cells in CRC is required.

### Interventions Manipulating Heterogeneity Within CD4^+^T Cell Functional Categories

T-bet, GATA3 and RORγt are key regulators of lymphocyte behavior. Interventions designed to modulate these factors could influence functional heterogeneity within multiple lineages simultaneously. They are potentially powerful therapeutic targets. TBET and RORγt are particularly important in CRC. Expression of T-bet in both effector and regulatory lineages correlates with enhanced tumor response and improved outcome. RORγt exhibits more nuanced effects. Effector and regulatory cells that express RORγt promote tumor progression. Co-expression with T-bet, however, confers potent anti-tumor activity. Interventions should be designed to promote activation of the T-bet transcriptional program and minimize the proportion of RORγt single-positive cells. Complete abrogation of RORγt, however, could prove counterproductive. A balance may have to be found.

The mechanisms by which to exert this pressure must also be determined. TGF-β is one potential source of influence. It promotes Treg differentiation, type III (RORγt-mediated) inflammation, and inhibits TBET. Empiric evidence indicates potential utility. Elevated TGF-β is a marker of poor prognosis in CRC (172). Upregulation of Smad7, a negative mediator of TGF-β signaling, drives accumulation of TBET+ Th17 cells and improves tumor responses in a mouse model of CRC (136). Furthermore, antibody-mediated inhibition of TGF-β signaling in a mouse model of CRC promotes a rapid and long lasting Th1 response far more potent than checkpoint inhibition and capable of preventing metastasis (173). In mice with pre-existing metastases, TGF-β blockade renders tumors susceptible to checkpoint inhibition. Disruption of TGF-β signaling is an excellent candidate for therapeutic intervention in CRC.

The IL-6/STAT3 pathway is another promising target. IL-6 favors RORγt and is aberrantly activated in many tumor microenvironments. Myeloid-derived soluble IL-6 receptor can blunt Th1 and CD8 responses (174, 175). Concurrent inhibition of IL-6 and PD-1 leads to elevated Th1 levels and enhances response to checkpoint blockade in multiple mouse models (176, 177). Blockade of IL-6 signaling may yield similar effects in CRC. Pharmacologic inhibition of SIRT1, required for dimerization of STAT3 downstream of IL-6, reduces Th17 numbers in CRC patients and tumor development in mice (178). Care must be taken, however, to examine potential effects on dual Tbet+ RORγt+ cells when blocking this pathway.

IL-23, which also signals through STAT3, promotes tumorigenic Th17 cell differentiation in CRC. Blockade of IL-23 may therefore blunt pathogenic Th17 differentiation and, as with STAT3 inhibition, redirect developing cells to a Th1-like phenotype. But IL-23 is a member of the IL-12 family of cytokines and can promote IFN-*γ* production in Th17 cells *via* STAT4. Interference with this pathway also has the potential for unintended consequences.

Direct administration of IL-12 can promote type I (TBET-mediated) responses. When administered to mice harboring a toxigenic strain of *B. fragilis*, IL-12 monotherapy leads to increased tumor CTL numbers, though no change in tumor burden was seen. Co-administration of IL-10 also reduces tumor Th17 numbers, and dramatically improves tumor burden (167). This cooperative effect is promising, and suggests additional interactions could be similarly exploited. But its mechanism is incompletely understood, and it is difficult to anticipate which additional combinations will prove beneficial.

Selective amplification of Tfh may represent an alternative potential therapeutic avenue. Given its role in Tfh development, ICOS stimulation may promote accumulation of Tfh-like cells and development of ectopic lymphoid structures in CRC. ICOS levels correlate with survival in CRC, while its expression is reduced in distant metastases (179). ICOS ligation may additionally modulate the effector response. Intratumor ICOS+ T cells exhibit elevated TBET and IFN-*γ* expression, and ICOS-based chimeric antigen receptor T cells generate anti-tumor bipolar TBET+ RORγt+ effectors cells (179, 180).

Exploitation of Treg biology represents one of the most promising mechanisms for combatting CRC. Tumors can be classified into two groups based on the relative abundance of FOXP3-high and FOXP3-low eTregs. Infiltration by FOXP3-low eTregs confers significantly better prognosis (72). Conversion of FOXP3-high eTreg cells to pro-inflammatory FOXP3-low eTregs would release the pressure pro-inflammatory cells and potentiate interventions design to promote them. Unfortunately, very little is known about the signaling and transcriptional events that guide this transition. Both IL-12 and TGF-β are elevated in CRC tissue infiltrated by FOXP3-low eTregs, suggesting these factors could promote acquisition of a pro-inflammatory phenotype. Augmentation of IL-12 signaling may therefore benefit Treg responses as well, but enhanced TGF-β signaling may have undesirable effects on the balance of Th17 and Th1 cells, and could potentially increase total Treg numbers. Similarly, BLIMP1 has been shown to prevent production of inflammatory cytokines in RORγt+ Treg cells. But inhibition of BLIMP1 would be expected to have deleterious effects on the effector response. As with other proposed interventions, targeted approaches localizing effects to specific cell populations might be required. Bi-specific antibodies, for example, could be used to block signaling events in specific subsets of T cells, including Tregs. Even so, these interventions are highly speculative. Our understanding of eTreg cell states is limited. The molecular determinants guiding their development must be elucidated before viable interventions can be developed.

### Manipulation of the Colonic Microbiota

Tumors preferentially develop in the distal colon and rectum, which harbors the highest concentration of microbial species (181). Early studies using germ free animals confirmed a role for microbial organisms in the development of CRC (182). 16S rRNA sequencing has identified differences in fecal and tumor mucosal microbiota between CRC patients and healthy controls (183). This dysbiosis is transferable, as fecal transplantation from tumor-bearing mice to conventionalized germ-free mice results in increased colon inflammation and tumorigenesis (184). Fecal transplants from CRC patients into germ-free mice also results in increased tumor burden (185). Interestingly, microbial patterns and signatures vary substantially between colon cancer tissue and adjacent non-malignant colon tissues (186). Thus, localized dysbiosis of intestinal microbiota can trigger inflammation leading to an increased permeability of the epithelial barrier and enhanced bacterial translocation, which in turn, promotes chronic inflammation by provoking a persistent immune response. This generates reactive oxygen and nitrogen species that lead to oxidative stress, DNA damage, and abnormal cellular proliferation, eventually culminating in the development of CRC.

While disparities between studies preclude the identification of a CRC-specific microbiome, substantial evidence supports causal roles for some species, including *Fusobacterium nucleatum* and *Bacteroides fragilis*. *Fusobacterium* is enriched in human CRC mucosa, predicts poor response to chemotherapy and prognosis and promotes tumor development in mice (183). Colonization persists even in distal metastases (187). Toxigenic *Bacteroides fragilis* is also enriched in CRC lesions, and promotes tumor development in mice. Interestingly, while toxigenic strains of *B. fragilis* promote tumor development, non-toxigenic strains confer protection by promoting infiltration of Tfh and development of ectopic lymphoid structures (137, 141).

Interventions should be designed to alter microbial populations to promote a beneficial immune response. Due to the localized nature of dysbiosis, direct sampling of colonic mucosa may be required to identify relevant organisms. Species level identification may not be sufficient given the strain dependent effects of *B. fragilis*. In addition, commensal organisms form a complicated, inter-dependent network. Manipulations affecting single species could prove insufficient to alter function. More sophisticated approaches should be considered. The potential therapeutic utility is apparent but, as before, our ability to exploit this potential is hampered by an abridged appreciation of biology.

## Conclusion & Perspective

The gastrointestinal (GI) tract is a large surface lined by a single layer of epithelium exposed to trillions of microbes and innocuous substances from the diet. It harbors the largest collection of immune cells in the body. The gut immune system maintains a state of dynamic equilibrium, monitoring luminal contents to sustain tolerance to dietary and commensal antigens while retaining the ability to rapidly respond to invading pathogens. CD4^+^ T cells are essential for both arms of this delicate balancing act. In recent years, increasing awareness of the diversity of CD4^+^ T cell form and function, and the relationships between these cells, has exposed limitations to the established paradigm. Many fundamental questions will have to be addressed before a new model can be developed. The increasing complexity of lineage diversity and functional heterogeneity have made these questions harder to answer. But they must be answered. CD4^+^ T cells are a tremendously powerful tool. It will be very difficult to wield this tool for clinical benefit without understanding how it works.

A deeper understanding of the intersection between CD4^+^ T cells and CRC is also needed. What underlies the seemingly contradictory roles played by some cells? Both nTregs and pTregs are beneficial in controlling the inflammation that serves as the nidus for CRC, but are harmful after inflammation leads to cancer. And yet some Tregs shed their suppressive role, become eTregs, and participate in anti-cancer immune responses, much as effector cells do. Similarly, Th17 and Th22 cells promote pathogen clearance and epithelial barrier function, respectively. Effective clearance and barrier integrity minimize exposure of epithelial cells to noxious inflammatory stimuli. But the sustained activity of these cells promotes tumor development. In contrast, Th17 cells that also express TBET are an important component of anti-cancer responses. Similarly, the concerted influence of follicular T cells and the colonic microbiota can both promote and oppose CRC. The development of these populations, and their influence on inflammatory responses to CRC, must be resolved in greater detail so that they can be exploited to improve disease outcomes.

Regardless of the target, interventions must be designed with pleiotropic, combinatorial effects in mind. Independent effects on both effector and regulatory cell populations must be examined carefully. Potential effects on follicular T cells should also be considered, as should interactions with innate, epithelial and tumor cells. Given potentially counterproductive effects on disparate cell types, targeted interventions may afford enhanced efficacy.

In summary, the manipulation of CD4^+^T cells represent a potentially powerful tool in CRC. Current attempts are limited by an incomplete understanding of the underlying biology. A more nuanced understanding of lineage diversity and plasticity in inflammatory responses during CRC is needed. The contributions of specific cell populations must be better delineated to understand the best way to implement therapeutic approaches. The relationships between these cells, and the molecular determinants guiding their development, must be understood. Much remains to be done. But we are close enough to see the reward far outweighs the cost.

## Author Contributions

DD and RB contributed equally. All authors contributed to the article and approved the submitted version.

## Funding

This study has been supported by a Career Development Award grant to RB (Corresponding author) from Crohn’s and Colitis Foundation of America (Identifier# 347717) and by start-up funds from the University of Alabama School of Medicine (to RB).

## Conflict of Interest

The authors declare that the research was conducted in the absence of any commercial or financial relationships that could be construed as a potential conflict of interest.

## Publisher’s Note

All claims expressed in this article are solely those of the authors and do not necessarily represent those of their affiliated organizations, or those of the publisher, the editors and the reviewers. Any product that may be evaluated in this article, or claim that may be made by its manufacturer, is not guaranteed or endorsed by the publisher.
